# 1′′-Benzyl-1′-methyl-4′-(naphthalen-1-yl)naphthalene-2-spiro-3′-pyrrolidine-2′-spiro-3′′-indoline-1,2′′-dione

**DOI:** 10.1107/S1600536811021908

**Published:** 2011-06-18

**Authors:** S. Selvanayagam, B. Sridhar, P. Saravanan, R. Raghunathan

**Affiliations:** a Department of Physics, Kalasalingam University, Krishnankoil 626 126, India; b Laboratory of X-ray Crystallography, Indian Institute of Chemical Technology, Hyderabad 500 007, India; c Department of Organic Chemistry, University of Madras, Guindy Campus, Chennai 600 025, India

## Abstract

In the title compound, C_38_H_32_N_2_O_2_, the pyrrolidine ring adopts an envelope conformation, whereas the cyclo­hexa­none ring in the tetra­hydro­naphthalene fused-ring system adopts a half-chair conformation. The benzyl ring is oriented at an angle of 67.1 (1)° with respect to the naphthyl ring system. Four intra­molecular C—H⋯O close contacts and C—H⋯π inter­action are observed. In the crystal, mol­ecules associate via C—H⋯O hydrogen bonds, forming a *C*(12) chain motif along the *ac* plane.

## Related literature

For general background to pyrrolidine derivatives, see: Mendoza *et al.* (2011 ▶); Morais *et al.* (2009 ▶); Pettersson *et al.* (2011 ▶); Shi *et al.* (2011 ▶). For a related structure, see: Selvanayagam *et al.* (2011 ▶). For the superposition of related structures, see: Gans & Shalloway (2001 ▶). For ring-puckering parameters, see: Cremer & Pople (1975 ▶); Nardelli (1983 ▶).
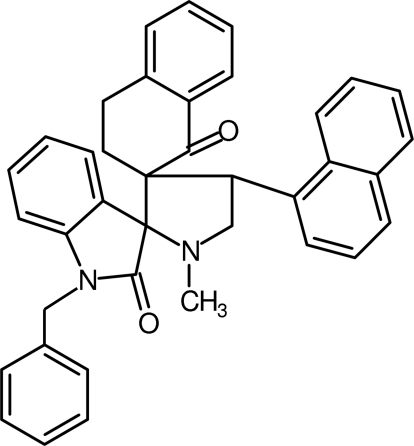


## Experimental

### 

#### Crystal data

C_38_H_32_N_2_O_2_
*M* *_r_* = 548.66
Monoclinic, 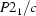

*a* = 12.6084 (6) Å
*b* = 14.3751 (7) Å
*c* = 17.4021 (9) Å
β = 110.057 (1)°
*V* = 2962.8 (3) Å^3^
*Z* = 4
Mo *K*α radiation
μ = 0.08 mm^−1^
*T* = 292 K
0.22 × 0.20 × 0.18 mm

#### Data collection

Bruker SMART APEX CCD area-detector diffractometer
7081 measured reflections
7081 independent reflections
4812 reflections with *I* > 2σ(*I*)

#### Refinement

*R*[*F* ^2^ > 2σ(*F* ^2^)] = 0.065
*wR*(*F* ^2^) = 0.149
*S* = 1.06
7081 reflections
380 parameters
H-atom parameters constrained
Δρ_max_ = 0.19 e Å^−3^
Δρ_min_ = −0.14 e Å^−3^

### 

Data collection: *SMART* (Bruker, 2001 ▶); cell refinement: *SAINT* (Bruker, 2001 ▶); data reduction: *SAINT*; program(s) used to solve structure: *SHELXS97* (Sheldrick, 2008 ▶); program(s) used to refine structure: *SHELXL97* (Sheldrick, 2008 ▶); molecular graphics: *ORTEP-3* (Farrugia, 1997 ▶) and *PLATON* (Spek, 2009 ▶); software used to prepare material for publication: *SHELXL97* and *PLATON*.

## Supplementary Material

Crystal structure: contains datablock(s) I, global. DOI: 10.1107/S1600536811021908/ng5180sup1.cif

Structure factors: contains datablock(s) I. DOI: 10.1107/S1600536811021908/ng5180Isup2.hkl

Additional supplementary materials:  crystallographic information; 3D view; checkCIF report

## Figures and Tables

**Table 1:** Hydrogen-bond geometry (Å, °) *Cg* is the centroid of the N2/C5/C1/C11/C6 ring.

*D*—H⋯*A*	*D*—H	H⋯*A*	*D*⋯*A*	*D*—H⋯*A*
C26—H26⋯O1^i^	0.93	2.54	3.399 (3)	154
C3—H3⋯O2	0.98	2.28	2.815 (2)	113
C4—H4*B*⋯O1	0.97	2.46	3.055 (2)	120
C10—H10⋯O2	0.93	2.53	3.182 (3)	127
C12—H12*A*⋯O1	0.97	2.38	3.078 (2)	128
C13—H13*A*⋯*Cg*	0.97	2.56	3.238 (2)	127

Symmetry code: (i) 
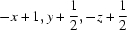
.

**Fig. 1.**
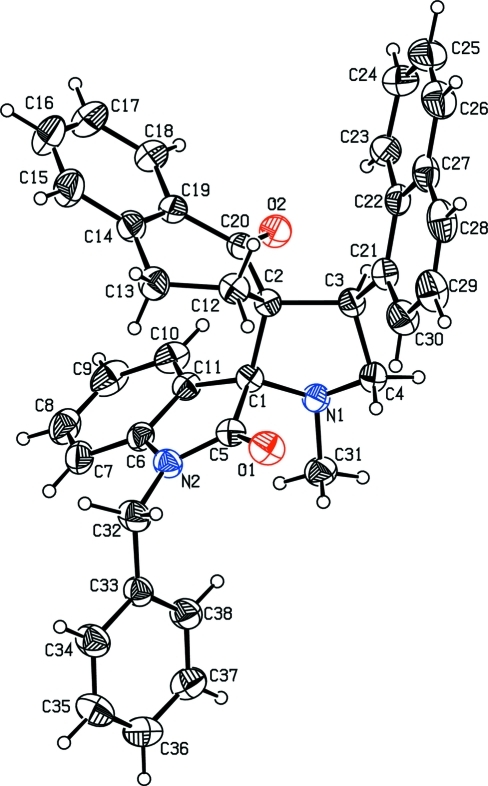
The molecular structure of the title compound, showing the atom-numbering scheme. Displacement ellipsoids are drawn at the 30% probability level

**Fig. 2.**
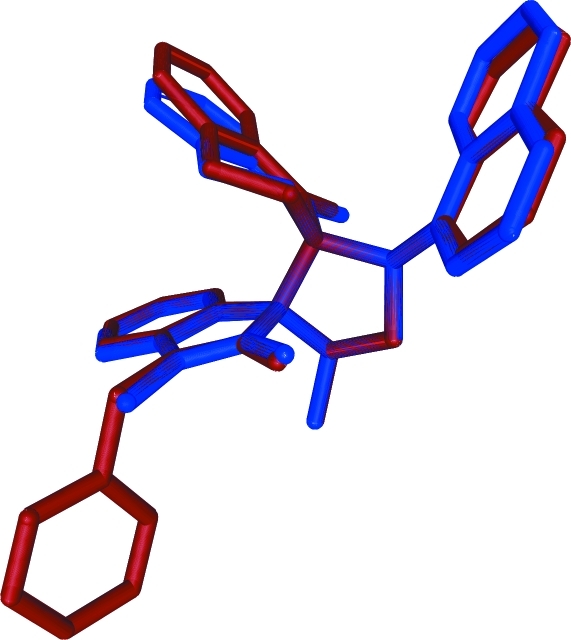
Superposition of (I) (red) with the similar reported structure of Selvanayagam et al. (2011) (blue).

**Fig. 3.**
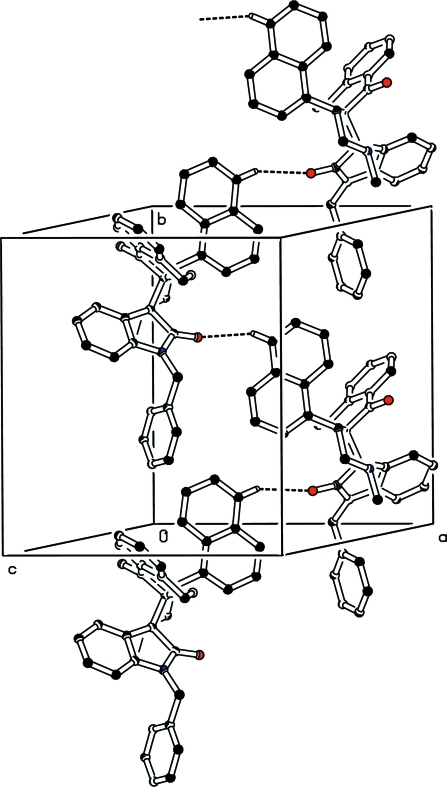
Molecular packing of the title compound, viewed along the c axis; H-bonds are shown as dashed lines forms a C(12) chain motif in unit cell. For the sake of clarity, H atoms, not involved in hydrogen bonds, have been omitted

## supplementary crystallographic information

### Comment

Pyrrolidine derivatives are dual norepinephrine reuptake inhibitors and 5-HT(2A) partial agonists (Pettersson *et al.*, 2011). These derivatives are also used as peptide deformylase inhibitors (Shi *et al.*, 2011). These derivatives possess anti-angiogenic (Morais *et al.*, 2009) and antimalarial (Mendoza *et al.*, 2011) activities. In view of these importance and continuation of our work on the crystal structure analyis of spiro-pyrrolidine derivatives, we have undertaken the crystal structure determination of the title compound, and the results are presented here.

The X-ray study confirmed the molecular structure and atomic connectivity for (I), as illustrated in Fig. 1. The geometry of all the ring systems (except benzyl ring) in the present structure is comparable with the related reported structure (Selvanayagam *et al.*, 2011). Fig. 2 shows a superposition of the pyrrolidine ring of (I) with this related reported structure, using Qmol (Gans & Shalloway, 2001); the r.m.s. deviation is 0.044 Å.

The sum of the angles at N1 of the pyrrolidine ring [335.3°] and N2 of the oxindole ring [360.1°] are in accordance with sp^3^ and sp^2^ hybridizations. The widening of the C21—C22—C23 and C21—C30—C29 bond angles [123.2 (2)° and 121.9 (2)°, respectively] are due to the short contacts H3···H23 (2 Å) and H4B···H30 (2.1 Å).

Pyrrolidine ring is in an envelope conformation, with puckering parameters q_2_ = 0.420 (2) Å and φ = 171.9 (3) °, and with atom C4 deviating -0.587 (2) Å from the least-squares plane passing through the remaining four atoms (N1/C1-C3) of that ring (Cremer & Pople, 1975). The cyclohexanone ring in the tetrahydro naphthalin ring system has a half-chair conformation with the lowest asymmetry parameters of ΔC_2_(C12-C13) = 0.093 (1)° (Nardelli, 1983). The mean plane of oxindole ring system make a dihedral angles of 44.0 (1) and 82.7 (1)°, respectively with respect to the naphtyl group systems and benzyl ring. The benzyl ring is oriented at an angle of 67.1 (1)° with respect to the naphthyl ring system.

The molecular structure is influenced by four intramolecular C—H···O close contacts and C—H···π interaction. Atoms O1 and O2 act as a bifurcated acceptor for these four C—H···O intramolecular close contacts. In the molecular packing, C—H···O hydrogen bonds involving atoms C26 and O1 link symmetry-related molecules to form C(12) chain motif in the unit cell. (Fig. 3 and Table 1).

### Experimental

To a mixture of N-Benzyl isatin (1mmol), sarcosine (1mmol) and 2-napthalidene- 1,2,3,4-tetrahydronaphthalene-1-ones (1mmol) was added and heated under reflux in methanol (20ml) until the disappearance of the starting materials as evidenced by TLC. The solvent was removed under vacuo. The crude product was subjected to column chromatography using petroleum ether-ethyl acetate as eluent. Single crystals were grown by slow evaporation from methanol.

### Refinement

H atoms were placed in idealized positions and allowed to ride on their parent atoms, with C—H distances of 0.93-0.97 Å, and Uiso(H) = 1.5U_eq_(C) for methyl H and Uiso(H) = 1.2U_eq_(C) for all other H atoms. During the structure analysis, it was observed that the unit cell contains large accesible voids in the crystal structure which tend to host unpredictable disordered solvent molecules. This affects the diffraction pattern, mostly a low scattering angles and was corrected with the SQUEEZE program (Spek, 2009).

### Crystal data

**Table:**

C_38_H_32_N_2_O_2_	*F*(000) = 1160
*M**_r_* = 548.66	*D*_x_ = 1.230 Mg m^−^^3^
Monoclinic, *P*2_1_/*c*	Mo *K*α radiation, λ = 0.71073 Å
Hall symbol: -P 2ybc	Cell parameters from 19526 reflections
*a* = 12.6084 (6) Å	θ = 2.4–28.3°
*b* = 14.3751 (7) Å	µ = 0.08 mm^−^^1^
*c* = 17.4021 (9) Å	*T* = 292 K
β = 110.057 (1)°	Block, colourless
*V* = 2962.8 (3) Å^3^	0.22 × 0.20 × 0.18 mm
*Z* = 4	

### Data collection

**Table:**

Bruker SMART APEX CCD area-detector diffractometer	4812 reflections with *I* > 2σ(*I*)
Radiation source: fine-focus sealed tube	*R*_int_ = 0.000
graphite	θ_max_ = 28.0°, θ_min_ = 1.7°
ω scans	*h* = −16→15
7081 measured reflections	*k* = 0→18
7081 independent reflections	*l* = 0→22

### Refinement

**Table:**

Refinement on *F*^2^	Primary atom site location: structure-invariant direct methods
Least-squares matrix: full	Secondary atom site location: difference Fourier map
*R*[*F*^2^ > 2σ(*F*^2^)] = 0.065	Hydrogen site location: inferred from neighbouring sites
*wR*(*F*^2^) = 0.149	H-atom parameters constrained
*S* = 1.06	*w* = 1/[σ^2^(*F*_o_^2^) + (0.0582*P*)^2^ + 0.6156*P*] where *P* = (*F*_o_^2^ + 2*F*_c_^2^)/3
7081 reflections	(Δ/σ)_max_ < 0.001
380 parameters	Δρ_max_ = 0.19 e Å^−^^3^
0 restraints	Δρ_min_ = −0.14 e Å^−^^3^

### Special details

**Table:**

Geometry. All esds (except the esd in the dihedral angle between two l.s. planes) are estimated using the full covariance matrix. The cell esds are taken into account individually in the estimation of esds in distances, angles and torsion angles; correlations between esds in cell parameters are only used when they are defined by crystal symmetry. An approximate (isotropic) treatment of cell esds is used for estimating esds involving l.s. planes.
Refinement. Refinement of F^2^ against ALL reflections. The weighted R-factor wR and goodness of fit S are based on F^2^, conventional R-factors R are based on F, with F set to zero for negative F^2^. The threshold expression of F^2^ > 2sigma(F^2^) is used only for calculating R-factors(gt) etc. and is not relevant to the choice of reflections for refinement. R-factors based on F^2^ are statistically about twice as large as those based on F, and R- factors based on ALL data will be even larger.

### Fractional atomic coordinates and isotropic or equivalent isotropic displacement parameters (Å^2^)

**Table:**

	*x*	*y*	*z*	*U*_iso_*/*U*_eq_	
O1	0.66021 (11)	0.12013 (10)	0.16760 (9)	0.0662 (4)	
O2	0.93723 (11)	0.40608 (9)	0.17479 (8)	0.0626 (4)	
N1	0.90128 (12)	0.19586 (10)	0.24061 (8)	0.0493 (4)	
N2	0.73609 (12)	0.08330 (10)	0.06975 (9)	0.0515 (4)	
C1	0.82372 (13)	0.20914 (12)	0.15647 (10)	0.0438 (4)	
C2	0.77474 (14)	0.31053 (12)	0.15932 (10)	0.0440 (4)	
C3	0.79927 (15)	0.32572 (12)	0.25429 (10)	0.0483 (4)	
H3	0.8622	0.3698	0.2731	0.058*	
C4	0.84428 (17)	0.23271 (13)	0.29382 (11)	0.0579 (5)	
H4A	0.8965	0.2410	0.3492	0.069*	
H4B	0.7833	0.1922	0.2949	0.069*	
C5	0.72848 (14)	0.13465 (12)	0.13340 (11)	0.0478 (4)	
C6	0.82563 (15)	0.11410 (13)	0.04566 (10)	0.0500 (4)	
C7	0.8606 (2)	0.07633 (15)	−0.01446 (12)	0.0656 (6)	
H7	0.8223	0.0270	−0.0467	0.079*	
C8	0.9545 (2)	0.11472 (18)	−0.02475 (14)	0.0774 (7)	
H8	0.9791	0.0918	−0.0658	0.093*	
C9	1.0130 (2)	0.18583 (18)	0.02375 (15)	0.0766 (7)	
H9	1.0775	0.2093	0.0161	0.092*	
C10	0.97681 (16)	0.22356 (15)	0.08476 (13)	0.0617 (5)	
H10	1.0168	0.2716	0.1179	0.074*	
C11	0.88083 (14)	0.18802 (12)	0.09463 (11)	0.0467 (4)	
C12	0.64951 (14)	0.32215 (14)	0.10675 (11)	0.0537 (5)	
H12A	0.6056	0.2749	0.1221	0.064*	
H12B	0.6235	0.3824	0.1180	0.064*	
C13	0.62867 (17)	0.31449 (15)	0.01543 (12)	0.0623 (5)	
H13A	0.6496	0.2528	0.0031	0.075*	
H13B	0.5490	0.3233	−0.0149	0.075*	
C14	0.69571 (18)	0.38566 (14)	−0.01072 (12)	0.0599 (5)	
C15	0.6569 (2)	0.42401 (19)	−0.08921 (14)	0.0832 (7)	
H15	0.5878	0.4050	−0.1263	0.100*	
C16	0.7195 (3)	0.4893 (2)	−0.11224 (16)	0.1014 (9)	
H16	0.6921	0.5144	−0.1647	0.122*	
C17	0.8221 (3)	0.51820 (19)	−0.05876 (17)	0.0964 (8)	
H17	0.8643	0.5621	−0.0750	0.116*	
C18	0.8620 (2)	0.48169 (15)	0.01912 (14)	0.0729 (6)	
H18	0.9314	0.5012	0.0555	0.087*	
C19	0.79923 (17)	0.41576 (13)	0.04390 (11)	0.0549 (5)	
C20	0.84519 (15)	0.38003 (12)	0.12938 (11)	0.0478 (4)	
C21	0.70292 (15)	0.36805 (13)	0.27567 (10)	0.0517 (4)	
C22	0.69140 (15)	0.46686 (14)	0.27729 (10)	0.0518 (4)	
C23	0.77083 (18)	0.52959 (14)	0.26515 (12)	0.0609 (5)	
H23	0.8326	0.5062	0.2537	0.073*	
C24	0.7590 (2)	0.62308 (16)	0.26981 (15)	0.0765 (6)	
H24	0.8134	0.6626	0.2628	0.092*	
C25	0.6657 (3)	0.66048 (18)	0.28506 (16)	0.0879 (8)	
H25	0.6581	0.7246	0.2879	0.106*	
C26	0.5871 (2)	0.60358 (19)	0.29565 (15)	0.0805 (7)	
H26	0.5245	0.6292	0.3045	0.097*	
C27	0.59733 (16)	0.50575 (16)	0.29370 (11)	0.0614 (5)	
C28	0.51909 (18)	0.4461 (2)	0.30987 (13)	0.0741 (6)	
H28	0.4570	0.4710	0.3199	0.089*	
C29	0.53320 (19)	0.3533 (2)	0.31101 (14)	0.0767 (7)	
H29	0.4823	0.3147	0.3237	0.092*	
C30	0.62455 (18)	0.31407 (16)	0.29314 (13)	0.0679 (6)	
H30	0.6318	0.2497	0.2932	0.082*	
C31	0.94324 (17)	0.10143 (13)	0.26161 (12)	0.0614 (5)	
H31A	0.8823	0.0617	0.2622	0.092*	
H31B	1.0009	0.1013	0.3147	0.092*	
H31C	0.9741	0.0791	0.2218	0.092*	
C32	0.65854 (17)	0.00833 (14)	0.03219 (13)	0.0641 (5)	
H32A	0.6339	0.0158	−0.0267	0.077*	
H32B	0.5924	0.0144	0.0483	0.077*	
C33	0.70527 (15)	−0.08882 (13)	0.05326 (11)	0.0532 (4)	
C34	0.64882 (19)	−0.16142 (15)	0.00444 (14)	0.0710 (6)	
H34	0.5845	−0.1495	−0.0406	0.085*	
C35	0.6867 (2)	−0.25106 (17)	0.02169 (16)	0.0816 (7)	
H35	0.6479	−0.2993	−0.0118	0.098*	
C36	0.7813 (2)	−0.27023 (16)	0.08771 (16)	0.0784 (7)	
H36	0.8065	−0.3312	0.0991	0.094*	
C37	0.83818 (19)	−0.19893 (16)	0.13677 (14)	0.0720 (6)	
H37	0.9024	−0.2113	0.1817	0.086*	
C38	0.80016 (17)	−0.10885 (14)	0.11940 (13)	0.0629 (5)	
H38	0.8393	−0.0607	0.1530	0.076*	

### Atomic displacement parameters (Å^2^)

**Table:**

	*U*^11^	*U*^22^	*U*^33^	*U*^12^	*U*^13^	*U*^23^
O1	0.0568 (8)	0.0683 (9)	0.0841 (10)	−0.0130 (7)	0.0380 (8)	−0.0036 (7)
O2	0.0576 (8)	0.0656 (9)	0.0608 (8)	−0.0172 (7)	0.0154 (7)	−0.0010 (7)
N1	0.0483 (8)	0.0490 (9)	0.0456 (8)	0.0023 (7)	0.0097 (7)	0.0001 (7)
N2	0.0499 (8)	0.0495 (9)	0.0508 (8)	−0.0032 (7)	0.0117 (7)	−0.0047 (7)
C1	0.0390 (9)	0.0460 (10)	0.0470 (9)	−0.0018 (7)	0.0155 (7)	−0.0007 (7)
C2	0.0414 (9)	0.0457 (10)	0.0443 (9)	−0.0004 (7)	0.0141 (7)	−0.0012 (7)
C3	0.0493 (10)	0.0500 (10)	0.0453 (9)	−0.0027 (8)	0.0160 (8)	−0.0040 (8)
C4	0.0690 (12)	0.0570 (12)	0.0454 (10)	0.0044 (10)	0.0167 (9)	0.0002 (9)
C5	0.0423 (9)	0.0468 (10)	0.0527 (10)	0.0004 (8)	0.0140 (8)	0.0012 (8)
C6	0.0523 (10)	0.0520 (11)	0.0436 (9)	0.0109 (9)	0.0140 (8)	0.0058 (8)
C7	0.0815 (15)	0.0681 (13)	0.0490 (11)	0.0195 (11)	0.0246 (11)	0.0034 (9)
C8	0.0981 (18)	0.0828 (17)	0.0657 (14)	0.0330 (15)	0.0466 (14)	0.0160 (13)
C9	0.0690 (14)	0.0885 (17)	0.0916 (17)	0.0188 (13)	0.0526 (13)	0.0307 (14)
C10	0.0527 (11)	0.0637 (13)	0.0761 (13)	0.0049 (9)	0.0317 (10)	0.0120 (10)
C11	0.0443 (9)	0.0488 (10)	0.0492 (9)	0.0069 (8)	0.0187 (8)	0.0056 (8)
C12	0.0433 (9)	0.0569 (11)	0.0580 (11)	0.0025 (8)	0.0134 (8)	−0.0015 (9)
C13	0.0527 (11)	0.0684 (13)	0.0542 (11)	0.0060 (10)	0.0033 (9)	−0.0027 (10)
C14	0.0675 (12)	0.0586 (12)	0.0504 (11)	0.0119 (10)	0.0160 (10)	−0.0013 (9)
C15	0.0942 (17)	0.0917 (18)	0.0537 (13)	0.0162 (14)	0.0124 (12)	0.0073 (12)
C16	0.135 (3)	0.105 (2)	0.0610 (15)	0.0160 (19)	0.0290 (17)	0.0285 (15)
C17	0.131 (2)	0.0851 (18)	0.0820 (18)	−0.0011 (17)	0.0482 (18)	0.0265 (15)
C18	0.0921 (16)	0.0630 (13)	0.0680 (13)	−0.0038 (12)	0.0330 (12)	0.0080 (11)
C19	0.0675 (12)	0.0478 (10)	0.0519 (10)	0.0057 (9)	0.0239 (10)	0.0016 (8)
C20	0.0507 (10)	0.0421 (9)	0.0504 (10)	0.0001 (8)	0.0169 (8)	−0.0024 (8)
C21	0.0510 (10)	0.0605 (12)	0.0443 (9)	−0.0029 (9)	0.0171 (8)	−0.0057 (8)
C22	0.0532 (10)	0.0608 (12)	0.0390 (9)	0.0068 (9)	0.0128 (8)	0.0000 (8)
C23	0.0669 (12)	0.0585 (12)	0.0603 (12)	0.0074 (10)	0.0257 (10)	0.0012 (9)
C24	0.0913 (17)	0.0583 (14)	0.0811 (15)	0.0064 (12)	0.0308 (13)	0.0062 (11)
C25	0.103 (2)	0.0639 (15)	0.0899 (18)	0.0290 (15)	0.0238 (15)	0.0018 (13)
C26	0.0754 (15)	0.0865 (18)	0.0754 (15)	0.0311 (14)	0.0203 (13)	−0.0077 (13)
C27	0.0520 (11)	0.0837 (15)	0.0439 (10)	0.0148 (10)	0.0106 (8)	−0.0056 (10)
C28	0.0530 (12)	0.111 (2)	0.0603 (13)	0.0049 (13)	0.0225 (10)	−0.0180 (13)
C29	0.0623 (13)	0.104 (2)	0.0727 (14)	−0.0181 (13)	0.0348 (12)	−0.0180 (13)
C30	0.0674 (13)	0.0735 (14)	0.0693 (13)	−0.0117 (11)	0.0315 (11)	−0.0112 (11)
C31	0.0615 (12)	0.0543 (12)	0.0606 (12)	0.0076 (9)	0.0108 (10)	0.0055 (9)
C32	0.0551 (11)	0.0584 (12)	0.0620 (12)	−0.0054 (9)	−0.0014 (9)	−0.0089 (9)
C33	0.0514 (10)	0.0530 (11)	0.0536 (10)	−0.0078 (9)	0.0157 (9)	−0.0054 (9)
C34	0.0669 (13)	0.0632 (14)	0.0726 (14)	−0.0075 (11)	0.0108 (11)	−0.0107 (11)
C35	0.0861 (17)	0.0606 (14)	0.0941 (17)	−0.0160 (13)	0.0257 (15)	−0.0181 (13)
C36	0.0867 (17)	0.0550 (13)	0.0996 (18)	0.0026 (12)	0.0399 (15)	0.0072 (12)
C37	0.0688 (14)	0.0680 (14)	0.0741 (14)	0.0012 (11)	0.0180 (11)	0.0129 (12)
C38	0.0624 (12)	0.0567 (12)	0.0615 (12)	−0.0075 (10)	0.0107 (10)	−0.0039 (10)

### Geometric parameters (Å, °)

**Table:**

O1—C5	1.221 (2)	C17—C18	1.378 (3)
O2—C20	1.218 (2)	C17—H17	0.9300
N1—C4	1.453 (2)	C18—C19	1.394 (3)
N1—C31	1.457 (2)	C18—H18	0.9300
N1—C1	1.468 (2)	C19—C20	1.490 (3)
N2—C5	1.362 (2)	C21—C30	1.370 (3)
N2—C6	1.404 (2)	C21—C22	1.429 (3)
N2—C32	1.451 (2)	C22—C23	1.416 (3)
C1—C11	1.517 (2)	C22—C27	1.426 (3)
C1—C5	1.555 (2)	C23—C24	1.358 (3)
C1—C2	1.590 (2)	C23—H23	0.9300
C2—C12	1.539 (2)	C24—C25	1.398 (3)
C2—C20	1.541 (2)	C24—H24	0.9300
C2—C3	1.589 (2)	C25—C26	1.346 (4)
C3—C21	1.515 (2)	C25—H25	0.9300
C3—C4	1.522 (3)	C26—C27	1.414 (3)
C3—H3	0.9800	C26—H26	0.9300
C4—H4A	0.9700	C27—C28	1.407 (3)
C4—H4B	0.9700	C28—C29	1.345 (3)
C6—C7	1.379 (3)	C28—H28	0.9300
C6—C11	1.390 (3)	C29—C30	1.409 (3)
C7—C8	1.372 (3)	C29—H29	0.9300
C7—H7	0.9300	C30—H30	0.9300
C8—C9	1.369 (3)	C31—H31A	0.9600
C8—H8	0.9300	C31—H31B	0.9600
C9—C10	1.401 (3)	C31—H31C	0.9600
C9—H9	0.9300	C32—C33	1.511 (3)
C10—C11	1.378 (2)	C32—H32A	0.9700
C10—H10	0.9300	C32—H32B	0.9700
C12—C13	1.523 (3)	C33—C38	1.376 (3)
C12—H12A	0.9700	C33—C34	1.379 (3)
C12—H12B	0.9700	C34—C35	1.371 (3)
C13—C14	1.494 (3)	C34—H34	0.9300
C13—H13A	0.9700	C35—C36	1.370 (3)
C13—H13B	0.9700	C35—H35	0.9300
C14—C19	1.394 (3)	C36—C37	1.369 (3)
C14—C15	1.397 (3)	C36—H36	0.9300
C15—C16	1.372 (4)	C37—C38	1.378 (3)
C15—H15	0.9300	C37—H37	0.9300
C16—C17	1.373 (4)	C38—H38	0.9300
C16—H16	0.9300		
			
C4—N1—C31	113.34 (15)	C16—C17—C18	119.5 (3)
C4—N1—C1	106.40 (13)	C16—C17—H17	120.2
C31—N1—C1	115.58 (14)	C18—C17—H17	120.2
C5—N2—C6	111.11 (14)	C17—C18—C19	120.6 (2)
C5—N2—C32	122.98 (16)	C17—C18—H18	119.7
C6—N2—C32	125.90 (16)	C19—C18—H18	119.7
N1—C1—C11	111.45 (13)	C18—C19—C14	119.87 (19)
N1—C1—C5	111.05 (13)	C18—C19—C20	118.38 (18)
C11—C1—C5	101.04 (13)	C14—C19—C20	121.75 (17)
N1—C1—C2	102.78 (13)	O2—C20—C19	119.97 (17)
C11—C1—C2	119.30 (14)	O2—C20—C2	120.77 (16)
C5—C1—C2	111.43 (13)	C19—C20—C2	119.26 (15)
C12—C2—C20	108.76 (14)	C30—C21—C22	118.20 (18)
C12—C2—C3	113.42 (14)	C30—C21—C3	121.82 (18)
C20—C2—C3	109.47 (13)	C22—C21—C3	119.98 (16)
C12—C2—C1	114.28 (14)	C23—C22—C27	117.36 (18)
C20—C2—C1	107.98 (13)	C23—C22—C21	123.22 (17)
C3—C2—C1	102.66 (13)	C27—C22—C21	119.40 (18)
C21—C3—C4	116.62 (15)	C24—C23—C22	121.6 (2)
C21—C3—C2	115.11 (14)	C24—C23—H23	119.2
C4—C3—C2	105.01 (14)	C22—C23—H23	119.2
C21—C3—H3	106.5	C23—C24—C25	120.6 (2)
C4—C3—H3	106.5	C23—C24—H24	119.7
C2—C3—H3	106.5	C25—C24—H24	119.7
N1—C4—C3	102.79 (14)	C26—C25—C24	120.0 (2)
N1—C4—H4A	111.2	C26—C25—H25	120.0
C3—C4—H4A	111.2	C24—C25—H25	120.0
N1—C4—H4B	111.2	C25—C26—C27	121.5 (2)
C3—C4—H4B	111.2	C25—C26—H26	119.2
H4A—C4—H4B	109.1	C27—C26—H26	119.2
O1—C5—N2	124.20 (16)	C28—C27—C26	121.7 (2)
O1—C5—C1	127.08 (16)	C28—C27—C22	119.3 (2)
N2—C5—C1	108.64 (15)	C26—C27—C22	119.0 (2)
C7—C6—C11	122.88 (18)	C29—C28—C27	120.6 (2)
C7—C6—N2	127.01 (18)	C29—C28—H28	119.7
C11—C6—N2	110.04 (15)	C27—C28—H28	119.7
C8—C7—C6	117.1 (2)	C28—C29—C30	120.5 (2)
C8—C7—H7	121.4	C28—C29—H29	119.8
C6—C7—H7	121.4	C30—C29—H29	119.8
C7—C8—C9	121.8 (2)	C21—C30—C29	121.9 (2)
C7—C8—H8	119.1	C21—C30—H30	119.0
C9—C8—H8	119.1	C29—C30—H30	119.0
C8—C9—C10	120.6 (2)	N1—C31—H31A	109.5
C8—C9—H9	119.7	N1—C31—H31B	109.5
C10—C9—H9	119.7	H31A—C31—H31B	109.5
C11—C10—C9	118.7 (2)	N1—C31—H31C	109.5
C11—C10—H10	120.7	H31A—C31—H31C	109.5
C9—C10—H10	120.7	H31B—C31—H31C	109.5
C10—C11—C6	118.91 (17)	N2—C32—C33	115.50 (15)
C10—C11—C1	131.72 (17)	N2—C32—H32A	108.4
C6—C11—C1	109.17 (15)	C33—C32—H32A	108.4
C13—C12—C2	112.77 (15)	N2—C32—H32B	108.4
C13—C12—H12A	109.0	C33—C32—H32B	108.4
C2—C12—H12A	109.0	H32A—C32—H32B	107.5
C13—C12—H12B	109.0	C38—C33—C34	118.27 (19)
C2—C12—H12B	109.0	C38—C33—C32	123.44 (17)
H12A—C12—H12B	107.8	C34—C33—C32	118.29 (17)
C14—C13—C12	110.80 (16)	C35—C34—C33	120.6 (2)
C14—C13—H13A	109.5	C35—C34—H34	119.7
C12—C13—H13A	109.5	C33—C34—H34	119.7
C14—C13—H13B	109.5	C36—C35—C34	120.7 (2)
C12—C13—H13B	109.5	C36—C35—H35	119.7
H13A—C13—H13B	108.1	C34—C35—H35	119.7
C19—C14—C15	118.5 (2)	C37—C36—C35	119.4 (2)
C19—C14—C13	120.00 (17)	C37—C36—H36	120.3
C15—C14—C13	121.5 (2)	C35—C36—H36	120.3
C16—C15—C14	120.8 (2)	C36—C37—C38	119.9 (2)
C16—C15—H15	119.6	C36—C37—H37	120.1
C14—C15—H15	119.6	C38—C37—H37	120.1
C15—C16—C17	120.8 (2)	C33—C38—C37	121.15 (19)
C15—C16—H16	119.6	C33—C38—H38	119.4
C17—C16—H16	119.6	C37—C38—H38	119.4
			
C4—N1—C1—C11	170.62 (14)	C12—C13—C14—C19	30.5 (2)
C31—N1—C1—C11	−62.61 (19)	C12—C13—C14—C15	−149.0 (2)
C4—N1—C1—C5	−77.56 (16)	C19—C14—C15—C16	0.4 (3)
C31—N1—C1—C5	49.21 (19)	C13—C14—C15—C16	180.0 (2)
C4—N1—C1—C2	41.70 (16)	C14—C15—C16—C17	0.3 (4)
C31—N1—C1—C2	168.47 (14)	C15—C16—C17—C18	−0.6 (4)
N1—C1—C2—C12	−143.34 (14)	C16—C17—C18—C19	0.1 (4)
C11—C1—C2—C12	92.80 (18)	C17—C18—C19—C14	0.7 (3)
C5—C1—C2—C12	−24.3 (2)	C17—C18—C19—C20	−178.7 (2)
N1—C1—C2—C20	95.51 (15)	C15—C14—C19—C18	−0.9 (3)
C11—C1—C2—C20	−28.35 (19)	C13—C14—C19—C18	179.53 (19)
C5—C1—C2—C20	−145.49 (14)	C15—C14—C19—C20	178.45 (18)
N1—C1—C2—C3	−20.09 (15)	C13—C14—C19—C20	−1.1 (3)
C11—C1—C2—C3	−143.95 (14)	C18—C19—C20—O2	−2.5 (3)
C5—C1—C2—C3	98.91 (15)	C14—C19—C20—O2	178.12 (18)
C12—C2—C3—C21	−12.2 (2)	C18—C19—C20—C2	177.21 (17)
C20—C2—C3—C21	109.49 (17)	C14—C19—C20—C2	−2.2 (3)
C1—C2—C3—C21	−136.00 (15)	C12—C2—C20—O2	156.02 (17)
C12—C2—C3—C4	117.47 (16)	C3—C2—C20—O2	31.6 (2)
C20—C2—C3—C4	−120.87 (15)	C1—C2—C20—O2	−79.4 (2)
C1—C2—C3—C4	−6.35 (17)	C12—C2—C20—C19	−23.7 (2)
C31—N1—C4—C3	−174.48 (14)	C3—C2—C20—C19	−148.10 (15)
C1—N1—C4—C3	−46.38 (17)	C1—C2—C20—C19	100.85 (17)
C21—C3—C4—N1	159.66 (15)	C4—C3—C21—C30	−31.4 (2)
C2—C3—C4—N1	30.92 (17)	C2—C3—C21—C30	92.3 (2)
C6—N2—C5—O1	−177.45 (17)	C4—C3—C21—C22	149.21 (17)
C32—N2—C5—O1	3.8 (3)	C2—C3—C21—C22	−87.09 (19)
C6—N2—C5—C1	−0.32 (19)	C30—C21—C22—C23	176.09 (18)
C32—N2—C5—C1	−179.08 (15)	C3—C21—C22—C23	−4.5 (3)
N1—C1—C5—O1	58.9 (2)	C30—C21—C22—C27	−2.4 (3)
C11—C1—C5—O1	177.17 (17)	C3—C21—C22—C27	177.04 (15)
C2—C1—C5—O1	−55.1 (2)	C27—C22—C23—C24	0.7 (3)
N1—C1—C5—N2	−118.17 (15)	C21—C22—C23—C24	−177.78 (19)
C11—C1—C5—N2	0.15 (17)	C22—C23—C24—C25	−1.5 (3)
C2—C1—C5—N2	127.90 (15)	C23—C24—C25—C26	0.3 (4)
C5—N2—C6—C7	177.43 (18)	C24—C25—C26—C27	1.5 (4)
C32—N2—C6—C7	−3.9 (3)	C25—C26—C27—C28	175.9 (2)
C5—N2—C6—C11	0.4 (2)	C25—C26—C27—C22	−2.2 (3)
C32—N2—C6—C11	179.10 (16)	C23—C22—C27—C28	−177.11 (17)
C11—C6—C7—C8	0.2 (3)	C21—C22—C27—C28	1.5 (3)
N2—C6—C7—C8	−176.48 (18)	C23—C22—C27—C26	1.0 (3)
C6—C7—C8—C9	1.6 (3)	C21—C22—C27—C26	179.63 (18)
C7—C8—C9—C10	−1.6 (3)	C26—C27—C28—C29	−177.2 (2)
C8—C9—C10—C11	−0.4 (3)	C22—C27—C28—C29	0.9 (3)
C9—C10—C11—C6	2.1 (3)	C27—C28—C29—C30	−2.3 (3)
C9—C10—C11—C1	176.24 (18)	C22—C21—C30—C29	1.1 (3)
C7—C6—C11—C10	−2.1 (3)	C3—C21—C30—C29	−178.35 (18)
N2—C6—C11—C10	175.08 (16)	C28—C29—C30—C21	1.3 (3)
C7—C6—C11—C1	−177.46 (16)	C5—N2—C32—C33	−105.3 (2)
N2—C6—C11—C1	−0.28 (19)	C6—N2—C32—C33	76.1 (2)
N1—C1—C11—C10	−56.4 (2)	N2—C32—C33—C38	17.9 (3)
C5—C1—C11—C10	−174.47 (19)	N2—C32—C33—C34	−163.17 (19)
C2—C1—C11—C10	63.1 (3)	C38—C33—C34—C35	0.0 (3)
N1—C1—C11—C6	118.11 (15)	C32—C33—C34—C35	−179.0 (2)
C5—C1—C11—C6	0.08 (17)	C33—C34—C35—C36	0.0 (4)
C2—C1—C11—C6	−122.36 (16)	C34—C35—C36—C37	0.0 (4)
C20—C2—C12—C13	53.7 (2)	C35—C36—C37—C38	0.0 (4)
C3—C2—C12—C13	175.74 (15)	C34—C33—C38—C37	−0.1 (3)
C1—C2—C12—C13	−67.0 (2)	C32—C33—C38—C37	178.8 (2)
C2—C12—C13—C14	−58.2 (2)	C36—C37—C38—C33	0.1 (3)

### Hydrogen-bond geometry (Å, °)

**Table:**

Cg is the centroid of the N2/C5/C1/C11/C6 ring.

**Table:**

*D*—H···*A*	*D*—H	H···*A*	*D*···*A*	*D*—H···*A*
C26—H26···O1^i^	0.93	2.54	3.399 (3)	154
C3—H3···O2	0.98	2.28	2.815 (2)	113
C4—H4B···O1	0.97	2.46	3.055 (2)	120
C10—H10···O2	0.93	2.53	3.182 (3)	127
C12—H12A···O1	0.97	2.38	3.078 (2)	128
C13—H13A···Cg	0.97	2.56	3.238 (2)	127

Symmetry codes: (i) −*x*+1, *y*+1/2, −*z*+1/2.
